# The Role of Piromelatine on Peripheral and Hippocampal Insulin Resistance in Rat Offspring Exposed to Chronic Maternal Stress

**DOI:** 10.3390/ijms25137022

**Published:** 2024-06-27

**Authors:** Natasha Ivanova, Milena Atanasova, Dora Terzieva, Katerina Georgieva, Jana Tchekalarova

**Affiliations:** 1 Institute of Neurobiology, Bulgarian Academy of Sciences (BAS), 1113 Sofia, Bulgaria; 2 Department of Biology, Medical University of Pleven, 5800 Pleven, Bulgaria; milenaar2001@yahoo.com; 3 Department of Clinical Laboratory, Medical Faculty, Medical University of Plovdiv, 4002 Plovdiv, Bulgaria; terzieva2006@yahoo.com; 4 Department of Physiology, Medical University of Plovdiv, 5800 Pleven, Bulgaria; kng@plov.net

**Keywords:** prenatal stress, melatonin analog, glucose, insulin, HOMA-IR, insulin receptor, GLUT4

## Abstract

Prenatal stress (PNS), which alters the hypothalamic-pituitary-adrenal axis function in the offspring, predisposes to insulin resistance (IR) in later life and is associated with numerous disorders, including cognitive and memory impairments. At present, our main goal is to assess the effects of chronic piromelatine (Pir) administration, a melatonin analogue, on PNS-provoked IR in the periphery and the hippocampus in male and female offspring. Pregnant Sprague–Dawley rats were exposed to chronic stress (one short-term stressor on a daily basis and one long-term stressor on a nightly basis) from the first gestation week until birth. Vehicle or Pir 20 mg/kg were administered intraperitoneally for 21 days. Plasma glucose, serum insulin levels, and the homeostasis model assessment of insulin resistance (HOMA-IR) were determined as markers of peripheral IR. For the hippocampal IR assessment, insulin receptors (IRs) and glucose transporter 4 (GLUT4) were examined. Prenatally stressed offspring of both sexes indicated enhanced plasma glucose and serum insulin concentrations, increased HOMA-IR, and decreased hippocampal GLUT4 only in male rats. The PNS-induced changes were corrected by chronic treatment with Pir. The present results suggest that the melatoninergic compound Pir exerts beneficial effects on altered glucose/insulin homeostasis in PNS-exposed offspring.

## 1. Introduction

Prenatal stress (PNS) causing hyperfunction of the maternal endocrine system can set off far-reaching endocrinological alterations and neurodevelopmental difficulties in the generation [1,2,3,4,5]. Likewise, prolonged exposure in utero to excessive glucocorticoids (GCs) levels deteriorates the offspring glucose/insulin metabolic profiles and outcomes in insulin resistance (IR) [6]. Insulin resistance, characterized by an abnormal biological response to insulin, alone as a pre-diabetic state or as type 2 diabetes mellitus (T2DM) and obesity syndromes, is a risk factor for metabolic complications and cognitive dysfunction [7,8,9]. Impaired insulin signaling with cognitive and memory deficiency has also been shown in patients without T2DM [10].

Clinically, the IR manifests with systemic hyperglycemia and compensatory hyperinsulinemia [11]. Glucose, the major energy supply for the body and the brain, can cross the blood-brain barrier and is translocated to the cell via glucose transporters [8]. Insulin, the primary regulator of glucose metabolism in the body, also enters the brain and exerts neuronal and hippocampal modulation [10]. Explorations indicate that elevated GCs have higher glycemic and hyperinsulinemic effects and impair insulin action [12,13]. Relatedly, the PNS alters glucose homeostasis through an abnormal hypothalamic-pituitary-adrenal (HPA) axis, resulting in hyperglycemia and compromised insulin signaling that triggers postnatal IR and complications later in life [14,15,16]. 

Insulin is very important for the brain, and deterioration of the brain’s insulin signaling negatively affects hippocampal plasticity, learning, and memory function [8]. Insulin receptors (IRs), abundant in the adipose, muscle tissues, and brain, are stimulated by insulin to activate the expression of the major insulin-dependent glucose transporter type 4 (GLUT4) [8]. The GLUT4 receptor in the brain is specifically expressed in the hippocampus, suggesting an important role for this receptor in hippocampal learning and memory functions [8,17]. The IR in the periphery, before the establishment of T2DM, may induce the development of hippocampal IR responsible for neuromental disorders [8]. Hippocampal IR is characterized by either downregulation of the IRs or, more commonly, post-receptor defects that affect GLUT4 expression and result in decreased glucose utilization [18]. Although the brain is a large consumer of glucose, impaired insulin signaling leads to higher glucose levels, which have a toxic effect on the brain and bring about cognitive and memory decline [18]. Studies established that GCs excess decreases IR activity and suppresses the expression of GLUT4, thus contributing to IR development not only in the body but also in the brain, where it generates memorization dysfunction [19,20]. 

The authors suggest a reciprocal relationship between HPA axis hyperfunction and IR induction, both of which are associated with numerous prevalent diseases, including degenerative disorders and memory decline [21,22]. In our previous study, we reported that PNS-induced alterations in the feedback mechanism of the HPA axis and increased expression of hippocampal GC receptors in the offspring led to memory impairment in adulthood [23]. The excess of GCs promoted by constant stress, in addition to a number of physiological changes, give rise to disruption in the glucose and insulin metabolisms, predisposing to IR and T2DM, accompanied by reduced cognition and dementia [21,22].

We and others have shown that the melatoninergic system can modulate HPA axis activity and hippocampal plasticity, memory, and cognition in offspring exposed to PNS [23,24]. Furthermore, the potent antioxidant melatonin has the ability to improve insulin secretion, normalize glucose levels, reduce IR, and slow the progression of cognitive dysfunction in patients with T2DM and Alzheimer’s disease (AD) [25,26].

The multimodal agent Piromelatine (Pir) with a chemical formulation (N-(2-[5-methoxy-1H-indol-3-yl]ethyl)-4-oxo-4H-pyran-2-carboxamide), concomitantly activating melatonin (MT) type 1, 2, and 3 receptors and serotonin (5-HT) type 1A and 1D receptors, is currently in a phase II clinical study for the treatment of insomnia in AD patients and is expected to improve mood, memory, cognition, and neurogenesis [27]. Our previous research in a generation with a history of maternal stress has demonstrated various beneficial effects of the drug on HPA axis activity, GCs receptors in the hippocampus, emotional behavior, sleep/wake cycles, as well as memory and cognitive functions [3,23,28]. Advantageous effects of Pir on metabolism have been reported by several studies. She et al. revealed that Pir increased insulin sensitivity and alleviated IR in rat models of a high-fat/sucrose diet and sleep deprivation [29,30]. Zhou et al. reported that in rats exposed to chronic stress in combination with a high-fat diet, the chronic Pir treatment improved glucose homeostasis and prevented whole-body IR [31]. Additionally, Li et al. attested that Pir refined the diabetic properties in rats [32]. Moreover, favorable, distinctive biological responses of Pir have been verified in insulin-resistant 3T3-L1 adipocytes [33,34].

Considering all these data, we hypothesize that PNS-induced peripheral and hippocampal IR in the offspring could be positively influenced by the melatoninergic compound Pir, something that could be behind the memory and learning improvement.

## 2. Results

### 2.1. The Effects of Chronic Pir Administration on Plasma Glucose Levels in Prenatally Stressed Male and Female Offspring

**Figure 1:**
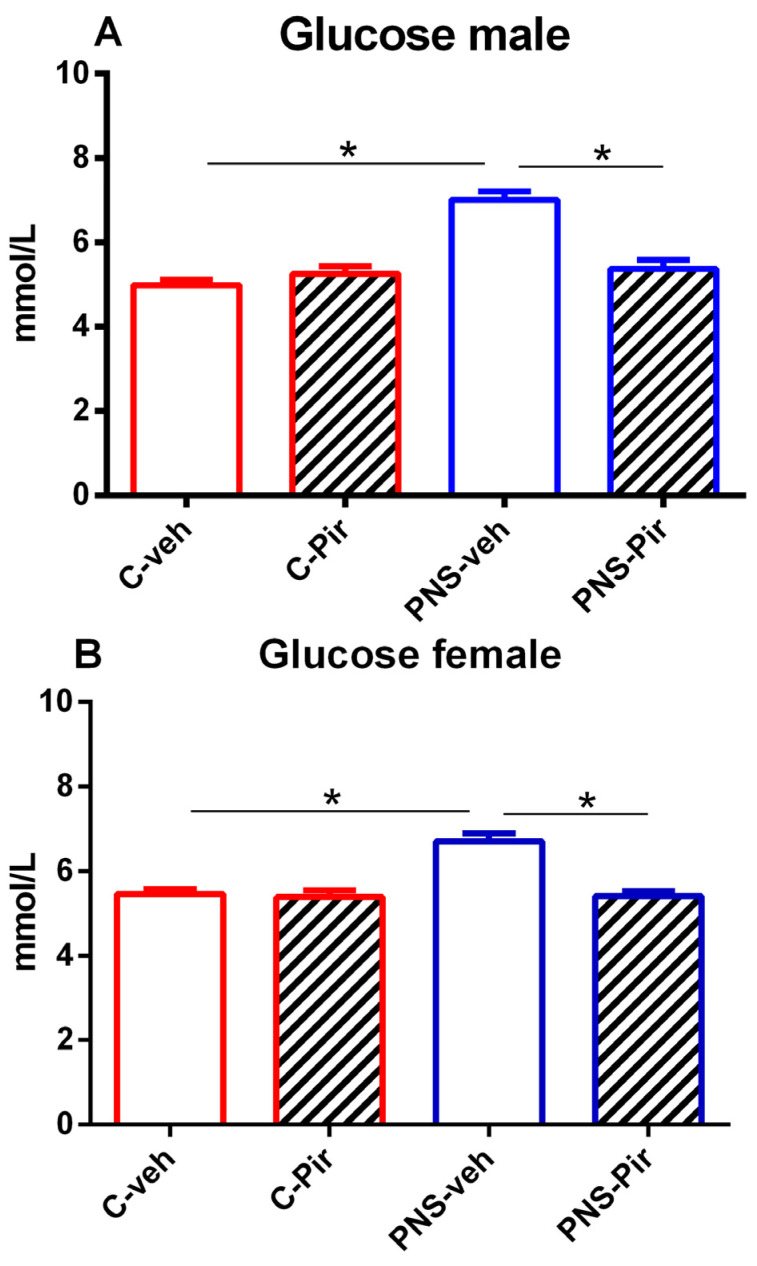
The chronic Pir administration produced a beneficial effect on plasma glucose levels in (**A**) male and (**B**) female offspring with a history of prenatal stress. Two-way ANOVA showed: (**A**) for the male group: a main effect of PNS [F_1,28_ = 36.385, *p* < 0.001], a main effect of Treatment [F_1,28_ = 15.041, *p* < 0.001] and PNS x Treatment interaction [F_1,28_ = 28.765, *p* < 0.001]; (**B**) for the female group: a main effect of PNS [F_1,28_ = 100.823, *p* < 0.001], a main effect of Treatment [F_1,28_ = 45.764, *p* < 0.001] and PNS x Treatment interaction [F_1,28_ = 32.035, *p* < 0.001]. Data are presented as means ± SEM: * *p* < 0.005 vs. C-veh, or PNS-veh.

The offspring of both sexes with a history of prenatal stress showed elevated plasma glucose levels compared with the controls (*p* < 0.05 PNS-veh vs. C-veh group) (Figure 1A,B). Chronic treatment with the melatonin analogue Pir was able to restore the normal glucose concentration in both male and female PNS offspring (*p* < 0.05 PNS-Pir vs. PNS-veh group) (Figure 1A,B).

### 2.2. The Effects of Chronic Pir Administration on Serum Insulin Levels in Prenatally Stressed Male and Female Offspring

**Figure 2:**
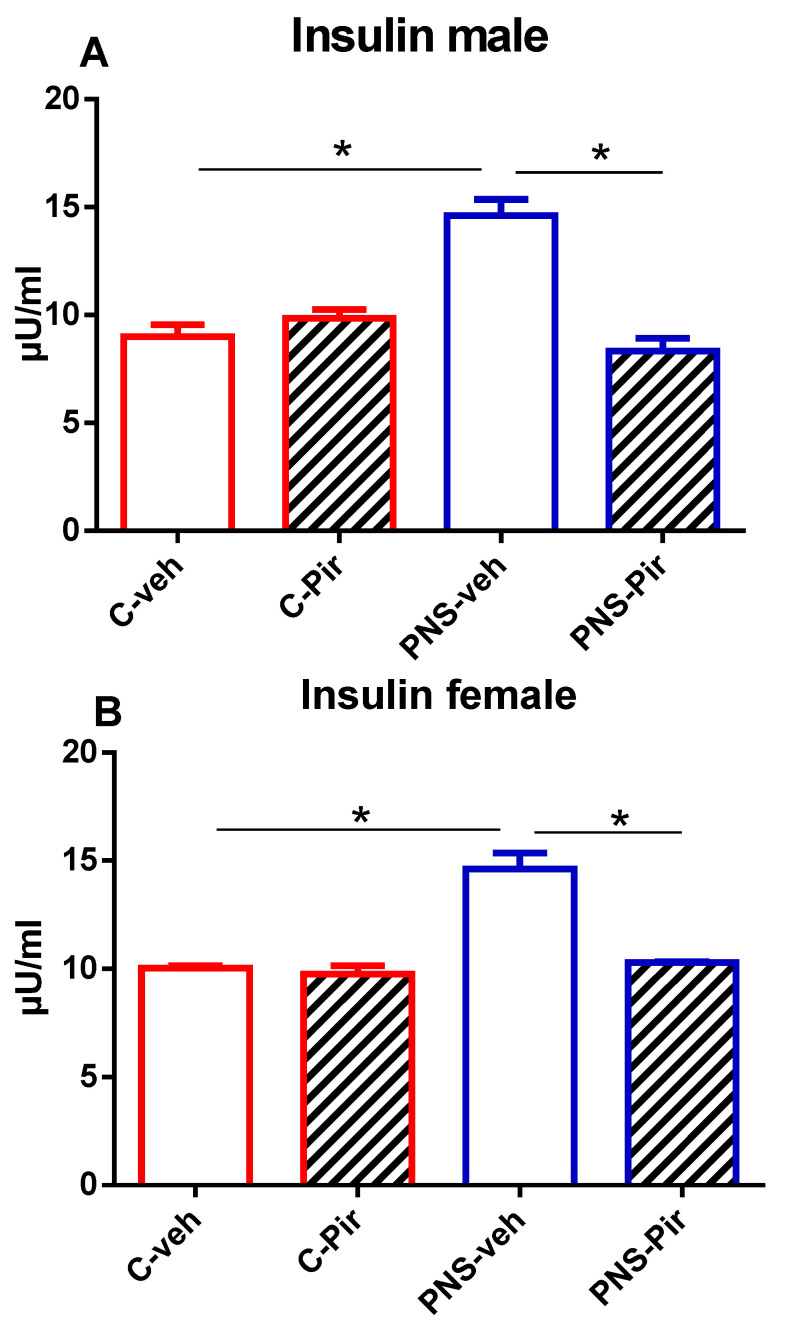
The chronic Pir administration produced a beneficial effect on serum insulin levels in (**A**) male and (**B**) female offspring with a history of prenatal stress. Two-way ANOVA showed: (**A**) for the male group: a main effect of PNS [F_1,28_ = 10.775, *p* < 0.001], a main effect of Treatment [F_1,28_ = 24.060, *p* < 0.001] and PNS x Treatment interaction [F_1,28_ = 35.183, *p* < 0.001]; (**B**) for the female group: a main effect of PNS [F_1,28_ = 36.960, *p* < 0.001], a main effect of Treatment [F_1,28_ = 29.838, *p* < 0.001] and PNS x Treatment interaction [F_1,28_ = 23.059, *p* < 0.001]. Data are presented as means ± SEM: * *p* < 0.005 vs. C-veh, or PNS-veh.

Similarly to the plasma gluose concentration, serum insulin levels were significantly increased in the prenatally stressed male and female groups compared with their matched controls (*p* < 0.05 PNS-veh vs. C-veh group) (Figure 2A,B). Piromelatine effectively reduced the serum insulin concentrations to normal levels in the male and female offspring exposed to prenatal stress (*p* < 0.05, PNS-Pir vs. PNS-veh group) (Figure 2A,B).

### 2.3. The Effects of Chronic Pir Administration on HOMA-IR in Prenatally Stressed Male and Female Offspring (Measurement of Fasting Plasma Glucose and Serum Insulin)

The HOMA-IR index was significantly increased in both the male and female PNS progeny, as demonstrated in Table 1. The chronic Pir treatment was able to reverse the abnormal HOMA-IR to control level in both PNS offspring sexes (Table 1).

### 2.4. The Effects of Chronic Pir Administration on GLUT4 Protein Levels in the Hippocampus of Prenatally Stressed Male and Female Offspring

**Figure 3:**
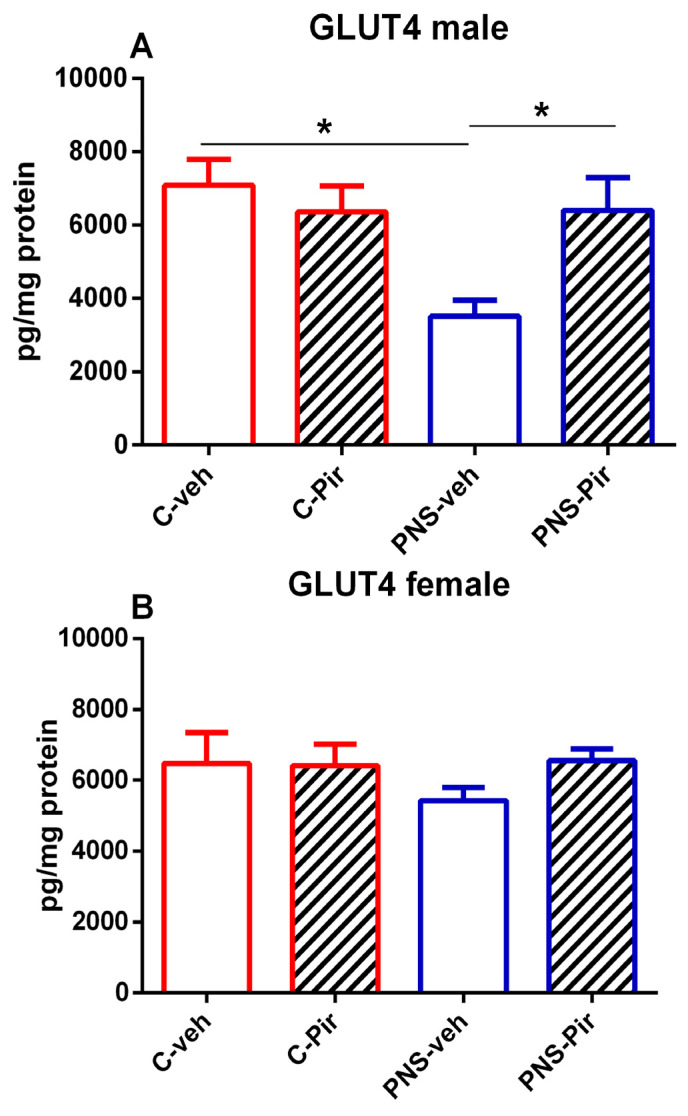
The chronic Pir administration produced a beneficial effect on hippocampal protein levels of GLUT4 in (**A**) PNS-male offspring. The levels of GLUT4 were not affected in the (**B**) female PNS offspring. Two-way ANOVA showed: (**A**) for the male group: a main effect of PNS [F_1,28_ = 6.277, *p* < 0.018] and PNS x Treatment interaction [F_1,28_ = 6.546, *p* < 0.016]. Data are presented as means ± SEM: * *p* < 0.005 vs. C-veh, or PNS-veh.

Exposure to PNS suppressed the GLUT4 in the hippocampus of the male offspring (*p* < 0.05 PNS-veh vs. C-veh group) (Figure 3A), while the hippocampal GLUT4 was not affected by the PNS in the female group (*p* > 0.05 PNS-veh vs. C-veh group) (Figure 3B). The MT compound Pir corrected the PNS-induced decrease in the hippocampal GLUT4 in the male group (*p* < 0.05 PNS-Pir vs. PNS-veh group) (Figure 3A).

### 2.5. The Effects of Chronic Pir Administration on IRs Protein Levels in the Hippocampus of Prenatally Stressed Male and Female Offspring

**Figure 4:**
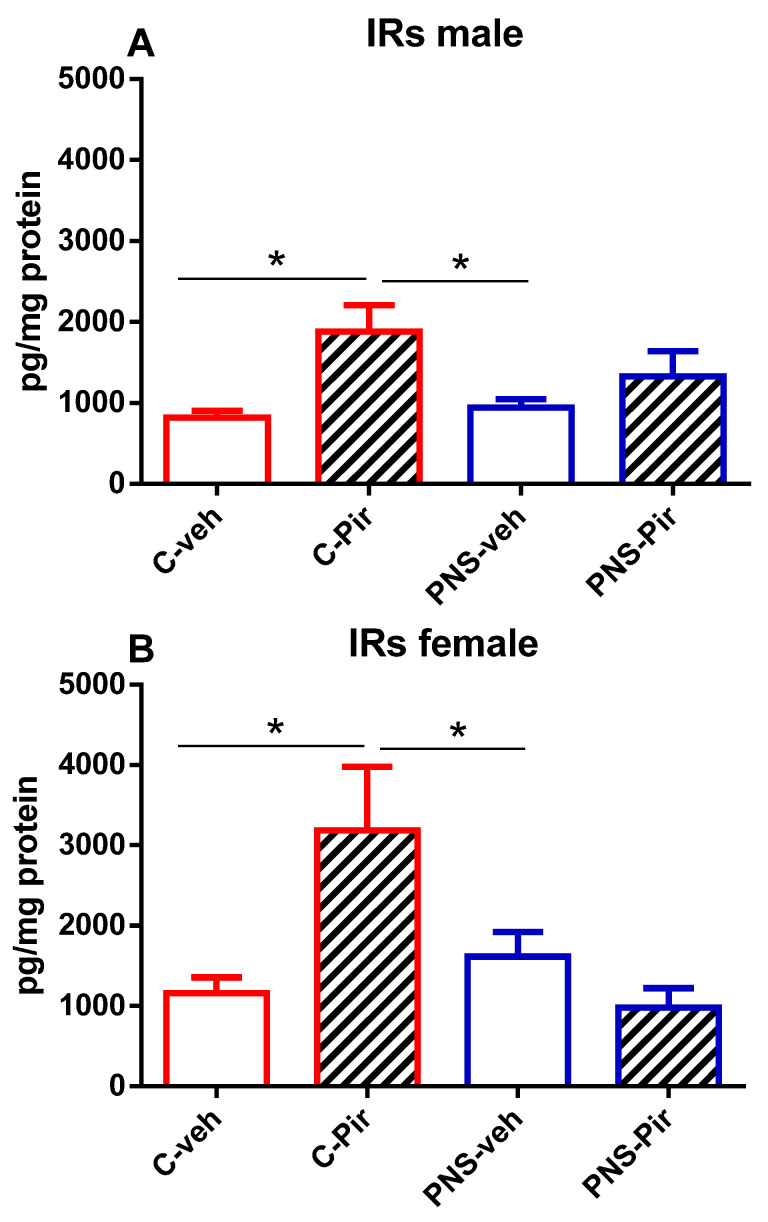
The chronic Pir administration produced a beneficial effect on hippocampal protein levels of IRs in the (**A**) control-male offspring and in the (**B**) control-female offspring with a history of PNS. Two-way ANOVA showed: (**A**) for the male group, a main effect of treatment [F_1,28_ = 9.429, *p* < 0.005]; (**B**) for the female group, a main effect of treatment [F_1,28_ = 8.649, *p* < 0.045]. Data are presented as means ± SEM: * *p* < 0.005 vs. C-veh or PNS-veh.

Hippocampal IRs in the offspring of both sexes were not affected by the PNS exposure (*p* > 0.05 PNS-veh vs. C-veh group) (Figure 4A,B). In the Pir-treated male and female control groups, the drug increased the hippocampal IRs compared with the vehicle-treated controls (*p* < 0.05, C-Pir vs. C-veh group) (Figure 4A,B).

## 3. Discussion

This study demonstrated the beneficial effect of the novel melatonin analogue Pir on IR by correcting systemic hyperglycemia and hyperinsulinemia in male and female offspring prenatally exposed to stress, as well as the ability of the drug to enhance the PNS-diminished GLUT4 expression in the hippocampus of male offspring.

We observed higher glucose concentrations in fasting plasma samples in PNS male and female rats. Consistently, other authors reported higher glycemia in adult male rats with PNS [14,35]. Another study showed hyperglycemia and glucose intolerance in aged male rats with PNS [36]. In a mouse model of prenatal restraint stress, males were hyperglycemic [6]. Similarly, several studies showed that in rats, including the Sprague–Dawley strain, overexposure to GCs in the fetal period, whether externally administered or maternal stress-induced, produces long-term hyperglycemia in offspring [37,38]. Undoubtedly, these data confirm that stress during pregnancy via increased GCs programs glucose homeostasis in adulthood and predisposes to metabolic and cognitive disorders [14,18]. In contrast, no differences in glucose metabolism were observed in the restraint PNS model of BALB/c mice or in Wistar rats with PNS after GCs treatment [6,39]. Franco et al. showed that the glucose concentrations were not affected by the PNS in either male or female offspring, whereas the PNS of the dexamethasone-treated mother impaired the glucose tolerance in the female progeny [40]. The latter suggests that glucose homeostasis depends on the type of PNS model, the species, the strain, and the sex. Whereas most of the researchers focused on investigating predominantly male rats, in this study we used Sprague–Dawley rats and PNS with diverse types of stressors, and both male and female offspring rats displayed hyperglycemia. The long-lasting changes in glucose metabolism are possibly mediated by the altered HPA axis feedback mechanism in the PNS offspring [5]. The potency of the melatoninergic substance Pir to regulate glucose levels, like melatonin, has been demonstrated in other studies with male rats: in high-fat or high-sugar diet models, sleep restriction, and chronically stressed rats combined with a high-fat diet, as well as in high-fat diet plus streptozotocin-treated rats [29,30,31,32]. We have shown for the first time that the drug can modulate glucose homeostasis not only in male but also in female rats in PNS conditions. The plausible effects of Pir are due to its high affinity for MT receptors and thus its ability to restore the abnormal activity of the neuroendocrine stress system, as we have shown in previous work [3]. This confirms that Pir can regulate the glucose metabolism of male and female rats with PNS and prevent metabolic dysfunction and glucotoxicity caused by PNS.

Our results showed that fasting serum insulin levels were elevated in both sexes with PNS. These data are consistent with those of Detka et al., who reported elevated plasma insulin in male PNS rats [35]. Hyperinsulinemic male and female offspring were raised from pregnant rats treated with external GCs [39]. Another researcher reported that the external administration of GCs during pregnancy did not only have a sex-specific effect with lower insulin levels in females but still increased this hormone in male and female offspring [40]. In contrast, insulin levels were not affected by GCs-induced PNS in male Sprague–Dawley rats or in male mice subjected to restraint stress [6,41]. Obviously, like glucose, insulin metabolism is affected by the rodent, the strain, the sex, and the PNS model. Our investigation revealed that PNS in Sprague–Dawley rats correlated with hyperinsulinemia in both male and female offspring rats, and the latter was normalized to control levels by chronic Pir administration. The protective effect of Pir on IR, similar to melatonin, was confirmed by decreased abnormal insulin levels in sleep-restricted rats and improved insulin sensitivity in obese rats and in chronically stressed and high-fat-fed rats [29,30,31]. Because the PNS offspring is vulnerable to stress, this results in hypercortisolemia, which is associated with basal and glucose-induced enhanced insulin secretion [12]. Taking into account the modulatory effects of melatonin on stress hormones [26,42], we assume that the melatonin analogue Pir exhibits its positive effects on programmed hyperinsulinemia through activation of the melatoninergic system and recovery of the HPA axis dysregulation, as previously shown [3].

The induction of systemic IR postpartum was demonstrated by an increased HOMA-IR index (measurement of fasting plasma glucose and serum insulin) as a consequence of PNS. These data are consistent with other studies showing that Pir was able to improve insulin sensitivity by lowering the HOMA-IR index in chronically sleep-deprived rats as well as in chronically stressed obese rats [30]. We validated that Pir was able to reverse the abnormal glucose and insulin concentrations as aforementioned and reduce the HOMA-IR to control levels. These data expressed the protective effect of the drug on IR in PNS offspring.

In general, the PNS disrupted the glucose and insulin metabolism and produced systemic IR that may affect brain structures and functions, especially the hippocampus, which has been shown to be very responsive to metabolic stress [43]. The adverse effect of the IR and the HPA axis disturbance on learning and memory areas in the brain has been explained [18,19].

In this study, we indicated that hippocampal IRs were not affected by PNS exposure in either sex. Although the hippocampal IRs were not changed, the PNS male offspring exhibited downregulation of the insulin-dependent GLUT4, whereas they remained unaffected in the female rats. Contradictory, other investigators did not observe any change in the GLUT4 in the hippocampus of PNS male rats, while they were decreased in the frontal cortex [13,35]. The dissimilarities could be due to different types of stressors or periods of application in the PNS model, but studies are few and unsatisfactory, and further investigations are needed to verify this hypothesis.

Nevertheless, in this study, the PNS reduced the hippocampal insulin-dependent GLUT4 in male rats. The explanation of the last is that the insulin signaling could be ineffective not only due to the law affinity of the insulin to the IRs, but the higher glucocorticoid fetal exposure could have altered the post-receptor signaling pathway, such as decreased IR phosphorylation, insulin receptor substrates, phosphatidylinositol 3-kinases, etc. Correspondingly, Piroli et al. denoted that one-week corticosterone treatment did not lessen the expression of the hippocampal IRs in rats but significantly decreased their activity, resulting in hippocampal and cognitive dysfunctions [19]. Trustworthy is that, in addition to insulin, other molecules such as BDNF and estrogen are also responsible for the translocation of GLUT4 on the plasma membrane, and their diminished levels are associated with memory deficiency [44]. In our earlier investigation, we demonstrated that the PNS decreased BDNF in the progeny of both sexes, which was corrected by the Pir predominantly in male rats and led to improved hippocampal function. However, PNS did not alter the hippocampus-dependent spatial memory in female rats, which might be due to a possible effect of the estrous cycle and the estrogen-linked GLUT4 translocation. Estrogen, as a lipophilic hormone, can penetrate the brain and has been shown to modulate synaptic plasticity and, hence, affect memory [45]. In support of this information, researchers have shown that the IR is linked with lower estrogen levels in males or either decreased or increased estrogen in females [46,47].

These data suggest that stress during the uterine period can induce long-lasting IR in the offspring adult life that may be crucial to the memory and learning processes. However, data regarding PNS-impaired brain insulin signaling and its impact on offspring hippocampal memorization structures are insufficient and warrant future study. In the present experiment, long-term administration of Pir successfully reversed the PNS-diminished hippocampal GLUT4 in male rats. We also previously showed the drug’s ability to enhance the associative memory in both male and female PNS offspring as well as the spatial memory in male progeny exposed to maternal stress [23]. This demonstrated the potency of the melatonin compound to ameliorate the PNS-induced peripheral and hippocampal IR and thus improve memory performance. An increase in GLUT4 could be a response to enhance cognitive functions or counteract post-receptor insulin signaling and associated cognitive impairments in PNS states. Of interest is also the effect of Pir per se on elevating IRs in the hippocampus of control male and female offspring, which deserves prospective investigations.

This study gives insights on the capacity of the potent MT1/2 activator Pir for the treatment of disturbed glucose/insulin metabolism and IR in PNS conditions. Still, these data emphasize the need for experimental and clinical studies to elucidate the mechanism of action of the drug and the promoted adaptation to changes in metabolic demands or conditions.

## 4. Materials and Methods

### 4.1. Animals

Adult 60-day-old Sprague–Dawley male and female offspring rats (Charles River Laboratories, Calco, Italy), weighing 200–250 g, were used in the experiment. The rats were accommodated in standard laboratory cages (3–4 per cage) at light–dark cycle (12/12), temperature of 21 ± 1 °C, a humidity 50–60%, and ad libitum intake of food and water. All animal procedures were conducted according to the Declaration of Helsinki Guiding Principles on Care and Use of Animals (DHEW Publication, Washington, DC, USA, NHI 80–23) and the European Communities Council Directives of 24 November 1986 (86/609/EEC). The current project was approved by the Bulgarian Food Safety Agency (#58000183).

### 4.2. Procedure for PNS

After one week of acclimatization, mature female rats were coupled with males for breeding. Gestational day 0 (E0) was defined as the appearance of a vaginal plug. The pregnant rats were divided into two groups (*n* = 5 per group): PNS and control (C). The control group was left undisturbed, while the PNS group was placed in a different room and subjected to different stressors described in a previous study [3]. Briefly, starting on the 7th day of gestation (E7), the pregnant animals were exposed to one short-term daily (crowding cage, forced swimming, wet bedding, social stressor, restraint stress) and one long-term (fasting, home cages tilted at 45°, continuous lighting, flashing multicolor lighting) overnight stressor, which continued until birth (postnatal day (*p*) 0). Litters with more than 8 pups (of similar sex ratios) were used.

### 4.3. Piromelatine Administration and Protocol Design

Sexually mature offspring rats of both sexes were injected once a day intraperitoneally at 4:00 p.m. for 21 days with Pir (kindly gifted by Neurim Pharmaceuticals Ltd., Tel Aviv, Israel) at a dose of 20 mg/kg, dissolved in hydroxyethyl cellulose 1%. The dose and regimen (2 h before the start of the dark phase) were defined as previously described [3]. The same regimen and route were used for the vehicle application to the C and the matched PNS groups. Rat pups were randomly assigned to 8 groups (males and females, respectively, *n* = 8 per group): C-veh–control group treated with vehicle, C-Pir–control group treated with piromelatine, PNS-veh–prenatally stressed group treated with vehicle, and PNS-Pir–prenatally stressed group treated with Pir.

### 4.4. Biochemical Methods

Twenty-four hours after the last piromelatine/vehicle injection, the rats were sacrificed following light anesthesia (CO_2_), and after decapitation, the brains were immediately removed, the hippocampus was dissected carefully, and the tissue was frozen on dry ice and stored at −20 °C until assay. In addition, fasting trunk blood samples were collected.

#### 4.4.1. Measurement of Serum Insulin and Plasma Glucose

From the trunk blood, the serum was clotted for 10–20 min (room temperature), while the plasma was collected in EDTA tubes and mixed for 20 min. Then the samples were centrifuged for 20 min at 2000–3000 rpm, and the supernatants were collected and stored at −20 °C until assayed. For the measurement of the glucose and insulin concentrations, rat-sensitive ELISA (enzyme-linked immunosorbent assay) kits were used (Rat Glucose ELISA kit cat. No. MBS1600616 and Rat Insulin ELISA kit cat. No. RAB0904), following the manufacturer’s instructions (MYBIOSOURCE, Southern California, San Diego, CA, USA). The concentrations of both indicators were measured at the Sirio S microplate reader. The measures were expressed as mmol/L for the glucose and as μU/mL for the insulin.

#### 4.4.2. HOMA-IR

The homeostasis model assessment insulin resistance (HOMA-IR) as a validated model for defining IR was estimated. The HOMA-IR was calculated by the formula: HOMA-IR = glucose (mmol/L) × insulin (μU/mL)/22.5, using the measurements of fasting plasma glucose and serum insulin.

#### 4.4.3. Measurement of GLUT4 and IRs in the Hippocampus

The hippocampi were stored at −20 °C until processed. After tempering, the tissue samples were homogenized in cold PBS buffer (pH 7.4), centrifuged at 11,000× *g* at 4 °C for 10 min, and the supernatant was collected. Hippocampal protein levels of IRs and glucose transporter GLUT4 were measured by rat-sensitive ELISA kits (Rat Insulin Receptor ELISA kit cat. No. E-EL-R1118, Wuhan Elabscience Biotechnology Co., Ltd., Wuhan, China, and Rat GLUT4 ELISA kit cat. No. MBS160852, MYBIOSOURCE, Southern California, San Diego, CA, USA) as per the manufacturer instructions. The measures were expressed as pg/mg protein.

### 4.5. Statistical Analysis

Two software programs were used for the statistical analyses (data presented as mean ± SEM): SigmaStat^®^ (version 11.0) and GraphPad Prism (version 6). The results were assessed by a two-way ANOVA with factors PNS and treatment, followed by Bonferroni’s t-test. In cases where the data were not normally distributed, a non-parametric analysis (Kruskal–Wallis on ranks) with a Mann–Whitney U test was applied for the group comparisons. A *p*-value of <0.05 was judged as significant.

## 5. Conclusions

The current experiment revealed the beneficial effect of the novel melatonin analog Pir on PNS-induced IR in male and female offspring. Pir normalized systemic hyperglycemia and hyperinsulinemia, thereby decreasing the HOMA-IR index in both offspring sexes with PNS and correcting the hippocampal GLUT4 reduction in male offspring. The data confirmed that Pir is involved in the regulation of the glucose/insulin metabolism of offspring exposed to maternal stress. We considered that the positive influence of Pir on PNS-evoked hippocampal and peripheral IR in offspring is one of the mechanisms for memory enhancement.

## Figures and Tables

**Table 1:** The chronic Pir administration reduced the HOMA-IR index in the male and female PNS offspring.

Group	Male	Female
C-veh	2.05 ± 0.13	2.43 ± 0.03
C-Pir	2.30 ± 0.10	2.32 ± 0.07
PNS-veh	4.58 ± 0.32 *	4.35 ± 0.25 *
PNS-Pir	1.97 ± 0.14 ^o^	2.47 ± 0.05 ^o^

Data are presented as means ± SEM: * *p* < 0.005 vs. C-veh, or ^o^ *p* < 0.005 vs. PNS-veh.

## Data Availability

The data are available from the authors upon request.
